# Agroindustrial Wastes as Alternative for Lipase Production by* Candida viswanathii* under Solid-State Cultivation: Purification, Biochemical Properties, and Its Potential for Poultry Fat Hydrolysis

**DOI:** 10.1155/2016/1353497

**Published:** 2016-09-20

**Authors:** Alex Fernando de Almeida, Kleydiane Braga Dias, Ana Carolina Cerri da Silva, César Rafael Fanchini Terrasan, Sâmia Maria Tauk-Tornisielo, Eleonora Cano Carmona

**Affiliations:** ^1^Bioprocess Engineering and Biotechnology, Federal University of Tocantins (UFT), Rua Badejós, Chácaras 69/72, Zona Rural, 77402-970 Gurupi, TO, Brazil; ^2^Environmental Studies Center (CEA), Universidade Estadual Paulista (UNESP), Avenida 24-A, 1515 Bela Vista, 13506-900 Rio Claro, SP, Brazil; ^3^Biochemistry and Microbiology Department, Bioscience Institute (IB), Universidade Estadual Paulista (UNESP), Avenida 24-A, 1515 Bela Vista, 13506-900 Rio Claro, SP, Brazil

## Abstract

The aims of this work were to establish improved conditions for lipase production by* Candida viswanathii* using agroindustrial wastes in solid-state cultivation and to purify and evaluate the application of this enzyme for poultry fat hydrolysis. Mixed wheat bran plus spent barley grain (1 : 1, w/w) supplemented with 25.0% (w/w) olive oil increased the lipase production to 322.4%, compared to the initial conditions. When olive oil was replaced by poultry fat, the highest lipase production found at 40% (w/w) was 31.43 U/gds. By selecting, yeast extract supplementation (3.5%, w/w), cultivation temperature (30°C), and substrate moisture (40%, w/v), lipase production reached 157.33 U/gds. Lipase was purified by hydrophobic interaction chromatography, presenting a molecular weight of 18.5 kDa as determined by SDS-PAGE. The crude and purified enzyme showed optimum activity at pH 5.0 and 50°C and at pH 5.5 and 45°C, respectively. The estimated half-life at 50°C was of 23.5 h for crude lipase and 6.7 h at 40°C for purified lipase. Lipase presented high activity and stability in many organic solvents. Poultry fat hydrolysis was maximum at pH 4.0, reaching initial hydrolysis rate of 33.17 mmol/L/min. Thus,* C. viswanathii* lipase can be successfully produced by an economic and sustainable process and advantageously applied for poultry fat hydrolysis without an additional acidification step to recover the released fatty acids.

## 1. Introduction

Lipase is an enzyme that catalyzes the hydrolysis and synthesis of esters formed by the linkage of glycerol and long-chain fatty acids. Owing to properties such as catalytic activity over a wide range of temperature and pH, substrate specificity, and enantioselectivity, lipase presents important industrial application [1]. The numerous applications are also due to other catalyzed reactions that differ from their natural physiological reaction. This versatility makes it the enzyme of choice for application in food, detergent, pharmaceutical, leather, textile, cosmetic, and paper industries [2].

Lipase can be produced in submerged and solid-state cultivation by many microorganisms such as bacteria, yeast, and filamentous fungi. The utilization of solid-state cultivation (SSC) for enzyme production requires previous evaluation of important aspects, such as selection of a suitable microorganism and substrate, optimization of process parameters, and isolation and further purification of the product [3]. SSC is defined as a process in which the substrate itself acts as carbon/energy source in the absence or near-absence of free water, employing natural or inert substrates used as solid support [4]. SSC is of special economic interest for developing countries such as Brazil, with abundance of biomass and agroindustrial wastes, which can be used as inexpensive raw materials [5]. Brazil is known for its great renewable resources such as agricultural and forestry wastes such as sugarcane bagasse, rice straw, wheat straw, oat hulls, and wood chips. The production of organic residues is about 597 million tons/year. Proper use of these wastes helps to minimize environmental and energy problems; furthermore, they can be used to obtain products with important applications in the pharmaceutical and food industry [6].

Several agroindustrial wastes have been evaluated for lipase production by microorganisms. Sugarcane bagasse was used for lipase production by* Yarrowia lipolytica* [7] and* Rhizopus homothallicus* [8],* Jatropha* cake by* Pseudomonas aeruginosa* [9], rice bran by* Aspergillus niger* [10], and babassu cake by* Penicillium simplicissimum* [11]. SSC is advantageous for lipase production due to higher activity levels, increased productivity, the extracellular nature of the produced enzyme, and increased stability to pH and temperature. In addition, it allows the construction of more compact reactors with less energy requirements, causing less damage to the environment [12]. Ideally, the solid materials should act as physical support, source of nutrients, and also appropriate inducer for enzyme production; however it is difficult to obtain all these features from a single substrate. This bottleneck can be overcome by using a combination of different substrates [13]. For example, SSC using mixed wheat bran and gingelly oil cake substrates increased the lipase production to 36.0% by* A. niger* MTCC 2594 and maximum activity corresponded to 384.3 U/grams of dry substrate (gds) after cultivation at 30°C for 72 h.

Many lipase types have been purified and biochemically characterized because their properties are very important for industrial applications. Microbial lipase usually presents high thermostability and pH stability, solvent tolerance, and high specificity for hydrolysis of long-chain unsaturated fatty acids [14, 15].

Poultry fat is a low cost feedstock, which could be incorporated in delicatessen meats and has a substantial nutritional value due to its high content of unsaturated fatty acids, especially monounsaturated ones, such as oleic acid (45–50%) [16]. Production of fatty acids by hydrolysis of natural oils and fats has been exploited using renewable raw materials; and it is considered an important industrial operation, since 1.6 million tons of fatty acids is produced worldwide every year by this process [17]. The products, fatty acids, and glycerol are basic materials for a wide range of applications. Fatty acids are used as a feedstock for the production of oleochemicals such as fatty alcohols, fatty amines, and fatty esters. These oleochemicals are used as lubricant grease, antiblock agent, plasticizers, emulsifiers, and also ingredient in the manufacture of soaps, detergents, and animal feed [18].

*Candida viswanathii* was isolated from wastewater of a Brazilian oil refinery (Replan/Petrobras, Paulínia, São Paulo, Brazil) and selected as the best lipase producer among 19 filamentous fungi and yeasts. In this study,* C*.* viswanathii* growing at high fat and hydrocarbon concentrations was observed. Lipase production was induced by long-chain fatty acids and triacylglycerol and the enzyme was effective in hydrolyzing triolein and olive oil [19]. In further study, the best parameters for lipase production by this yeast were observed in liquid Vogel's medium with 1.5% (w/v) olive oil as carbon source and 0.1% (w/v) soybean lecithin as emulsifier under agitation of 210 rpm, at 27.5°C and pH 6.0 [20]. The biochemical characterization revealed that the crude lipase produced in liquid medium was quite different from those usually produced by other* Candida* species. Optimal activity at acid pH of 3.5 suggests a new lipolytic enzyme for this genus and for yeasts in general. In addition, the crude lipase presented high stability in acid condition and up to 40–45°C, remaining active in the presence of organic solvents such as dimethyl sulfoxide (DMSO) and methanol and gum Arabic emulsifier. The aim of this study was to evaluate the lipase production by* C. viswanathii* in solid-state cultivation using agroindustrial wastes and crude poultry fat as substrate. Purification and biochemical characterization of the crude and purified lipase were carried out, and also poultry fat was further hydrolyzed by both enzyme forms.

## 2. Materials and Methods

### 2.1. Microorganism and Preinoculum

*C. viswanathii* strain is available in the Culture Collection of the Environmental Studies Center, CEA/UNESP, Brazil.* C. viswanathii* was cultivated on malt extract agar (MEA) for 3 days, at 28°C, for inoculum preparation. Liquid medium was prepared using Vogel medium [21], with 1.5% (w/v) olive oil and 0.2% (w/v) yeast extract as single carbon and nitrogen sources, respectively, according to previous established conditions [19]. Erlenmeyer flasks (125 mL) containing 25 mL of medium were inoculated with 1.0 mL of cells suspension (1 × 10^7^ cells/mL) and incubated at 28°C, 210 rpm for 24 h. Five milliliters of this suspension was used for inoculating the substrates for solid-state cultures.

### 2.2. Solid-State Cultivation

Cultures were performed in Erlenmeyer flasks (250 mL) containing 10 g of wheat bran (WB), cassava peel, barley spent grain (BSG), sugarcane bagasse, or citrus pulp (CP), which were previously washed with distilled water, dried until constant weight, and sieved (18 mesh). Nonsieved BSG and CP were incorporated into wheat bran to improve aeration of the substrates, constituting mixed substrate cultivation. Olive oil (25%, w/w) was used as initial carbon/inducer source to the substrates. Modified Vogel salts solution [19] prepared without nitrogen source was used to provide 50% (w/v) initial moisture. Flasks containing moisturized and supplemented substrates were autoclaved at 121°C, for 20 min. Cultures were carried out at 28°C for 5 days.

### 2.3. Enzyme Extraction

After cultivation 100 mL of distilled water was added to each flask and the mixture was incubated on a rotary shaker (250 rpm, 4°C) for 60 min. Then, the suspension was filtrated through a double layer gauze cloth and centrifuged (8000 ×g, 20 min, 4°C). The clear supernatant was used as source of crude extracellular lipase.

### 2.4. Lipase Activity

Lipase activity was assayed with p-nitrophenyl palmitate (p-NPP) as substrate [20]. p-NPP was dissolved in 0.5 mL of dimethyl sulfoxide and then diluted to 50 mM with 50 mM sodium phosphate buffer pH 7.0 containing 0.5% (w/v) Triton X-100. The hydrolysis of p-NPP was determined discontinuously at 37°C by measuring the released p-nitrophenolate (p-NP). After 5 min preincubation of 0.9 mL of the substrate, adding 0.1 mL of appropriately diluted enzyme sample started the reaction. The reaction was stopped at different intervals by heat shock (90°C, 1 min), followed by addition of 1 mL of saturated sodium tetraborate solution. The absorbance was measured at 405 nm (molar extinction coefficient for p-NP: 1.8 × 10^4^ M^−1^ cm^−1^). Controls were prepared without enzyme. One unit of enzyme activity was defined as the amount of enzyme that releases 1 *μ*mol of p-NP per min.

### 2.5. Protein Analysis

Protein was determined with Coomassie blue G-250 [22], using bovine serum albumin as standard.

### 2.6. Parametric Optimization of Lipase Production

#### 2.6.1. Carbon Supplementation

The substrates were supplemented with 25% (w/w) natural triacylglycerols, palm oil, soybean oil, corn oil, canola oil, sunflower oil, linseed oil, and babassu oil, or with wastes such as poultry fat, beef tallow, lard, and cooking oil. Poultry fat used for inducing lipase production was evaluated at 5, 10, 15, 20, 25, 30, 35, 40, 45, and 50% (w/w).

#### 2.6.2. Nitrogen Supplementation

The medium was supplemented with 5% (w/w) corn steep liquor, yeast extract, soy protein, whey powder, and cotton protein. Yeast extract was evaluated at 1.0, 1.5, 2.0, 2.5, 3.0, 3.5, 4.0, 4.5, and 5.0% (w/w).

#### 2.6.3. Temperature and Moisture

The effect of temperature on lipase production was verified by carrying out cultivation at 15, 20, 25, 30, 35, and 40°C.

The effect of initial moisture content of the cultures was evaluated by adding Vogel salts [19] without nitrogen sources in order to provide 20, 30, 40, 50, 60, and 70% (w/w) initial moisture.

### 2.7. Enzyme Purification

The crude extract was previously dialyzed against 0.02 M ammonium acetate buffer pH 6.9 (8 h, 3 changes, 4°C). The dialyzed extract was applied to a hydrophobic Octyl Sepharose column (HiPrep™ 16/10 Octyl Sepharose FF fast flow, GE Healthcare) previously equilibrated in the same buffer, at 2 mL/min flow rate. The column was washed with 50 mL of the same buffer and 3.0 mL fractions were collected. Elution of bound proteins was performed with 100 mL of a 0.0 to 1.0% (w/v) Triton X-100 linear gradient prepared in the same buffer. Fractions with lipase activity were pooled and sample purity was evaluated by SDS-PAGE. All purification procedures were carried out at 4°C.

### 2.8. Enzyme Characterization

#### 2.8.1. SDS-PAGE

The purified enzyme was previously treated with Calbiosorb™ adsorbent resin (Calbiochem®, San Diego, USA) to remove Triton X-100. The resin was equilibrated in 0.05 M ammonium acetate buffer pH 6.9 and loaded with the purified enzyme. Samples were incubated at 10°C for 45 min under slow stirring and then centrifuged (8500 ×g, 4°C, 20 min). The supernatant containing enzyme was submitted to electrophoresis. Even after treatment, residual Triton X-100 was still detected in the sample by reading absorbance at 280 nm.

SDS-PAGE was performed using 10% (w/v) polyacrylamide gels according to Hames [23]. Samples were previously treated with 8 M urea according to Lesuisse et al. [24]. Resolved protein bands were visualized after staining with 0.1% (w/v) Coomassie brilliant blue R-250 in methanol, acetic acid, and distilled water (4 : 1 : 5, v/v/v). This method was used to determine the molecular mass (MW) of the purified enzyme using appropriate standards.

#### 2.8.2. Optimum pH and pH Lipase Stability

Enzyme activity was measured at 37°C in different pH values using 0.05 M glycine-HCl buffer pH from 2.0 to 3.0 and McIlvaine buffer pH from 3.0 to 8.0. Stability to pH was carried out with the same buffers, except in pH from 8.6 to 10.0 in which 0.05 M glycine-NaOH buffer was used. Enzyme samples were (1 : 2, v/v) diluted in each buffer and incubated for 24 h at 10°C.

#### 2.8.3. Optimum Temperature and Thermal Stability

The optimum temperature was determined by measuring enzyme activity in temperatures from 20 to 70°C, in McIlvaine buffer pH 5.0. For thermal stability, the enzyme was incubated at 40, 45, 50, 55, and 60°C in McIlvaine buffer pH 5.0 in the absence of substrate, and the residual activity was determined in McIlvaine buffer pH 5.0 at 50°C.

#### 2.8.4. Effect of Organic Solvents

The effect of organic solvents on activity and stability of crude and purified lipase was evaluated using 10% (v/v) glycerol, DMSO, propylene glycol, methanol, acetonitrile, ethanol, acetone, 1-propanol, 2-propanol, n-butanol, toluene, xylol, n-hexane, and isooctane. The effect of organic solvents on the activity was verified by adding each solvent into the enzymatic reactions. Stability experiments were carried out in sealed flasks shaken at 200 rpm, for 2 h at 30°C. Residual activities were determined in McIlvaine buffer pH 5.0 at 50°C and expressed in relation to the control without any substance.

#### 2.8.5. Specificity for Substrate

Specificity was verified using 0.5 mM p-nitrophenyl acetate, p-nitrophenyl butyrate, p-nitrophenyl octanoate, p-nitrophenyl decanoate, p-nitrophenyl laurate, p-nitrophenyl myristate, p-nitrophenyl palmitate, and p-nitrophenyl stearate by performing enzyme assays in McIlvaine buffer pH 5.0 at 45°C and pH 5.5 at 50°C for the crude and purified lipase, respectively.

#### 2.8.6. Kinetic Parameters

The activity of purified lipase was assayed with p-nitrophenyl palmitate from 0.0 to 1.0 mM. The Michaelis-Menten constant (*K*_*m*_) and maximum reaction velocity (*V*_max_) were estimated from Lineweaver-Burk plot [25].

#### 2.8.7. Hydrolysis of Poultry Fat

Hydrolysis of poultry fat was developed at 50°C by titration of released fatty acids. The oils (10%, w/v) were emulsified in McIlvaine buffer pH 4.0, 6.0, and 8.0, containing 0.5% (w/v) Triton X-100. The reaction was started by adding 1 mL of enzyme to 5 mL of this emulsion, and then it was maintained for 96 h at 200 rpm orbital agitation. The reaction was interrupted by adding 16 mL of an acetone : ethanol solution (1 : 1, v/v) to the mixture. The released fatty acids were titrated to pH 11 with a 0.05 M NaOH solution. From these values, the degree of hydrolysis was calculated according to [18]

(1) $$\begin{array}{c} X=\frac{W\left(V-V_{0}\right)M}{10mf_{0}}, \end{array}$$

where *X* is degree of hydrolysis (%); *W* is the mean molecular weight of the fatty acids; *V* is the volume (mL) of NaOH solution used for titration of the sample; *V*_0_ is the volume (mL) of NaOH solution used for titration of the control; *M* is the molarity of NaOH solution; *m* is the weight of the sample (mL); and *f*_0_ is weight fraction of oil in the start of reaction.

The initial rate of reaction was calculated using the following equation:

(2) $$\begin{array}{c} r_{0}=\frac{10^{4}S_{0}}{W}{\left(\;\frac{dX}{dt}\;\right)}_{t=0}, \end{array}$$

where *r*_0_ is initial rate of hydrolysis (mmol/L/min); *S*_0_ is initial concentration of oil (g/L); (*dX*/*dt*)_*t*=0_ is slope of the degree of hydrolysis (*X*) versus time curve at *t*_0_. The average molecular weight of fatty acids in poultry fat was calculated as 277.3 kg/kmol.

## 3. Results

### 3.1. Selection of Agroindustrial Substrates and Olive Oil Supplementation

Solid-state cultivation of* C. viswanathii* yeast on agroindustrial wastes was evaluated for lipase production using wheat bran, barley spent grain, cassava peel, sugarcane bagasse, and citrus pulp, assessed in the absence and in the presence of olive oil as inducer (Table 1). When the substrates were individually evaluated without olive oil supplementation, the highest level of enzyme activity was observed with wheat bran; intermediate level was observed with barley spent grain and low or absent enzyme activity was observed with citrus pulp, sugarcane bagasse, and cassava peel. The low levels of lipase observed in the medium without olive oil indicated that this yeast used soluble nutrients for microbial growth and the matrixes as support. Mixtures of wheat bran plus barley spent grain and wheat bran plus citrus pulp were evaluated at different proportions since the presence of nonsieved barley spent grain and citrus pulp could increase the aeration of the system and consequently increase fungal growth and enzyme production. The use of mixed substrates slightly changed enzyme production, considering the mixture of wheat bran plus barley spent grain at 3 : 2 proportion (0.88 U/gds), although this activity still was lower than that observed in cultivation only with wheat bran (0.94 U/gds). In other mixtures, the lipase activity was still lower when compared to wheat bran. The initial supplementation of the substrates with olive oil improved lipase production and the highest activity was also verified with wheat bran (5.79 U/gds and 3.50 U/mg of protein). The use of mixed substrates and olive oil supplementation also increased the activity and the highest production was observed with wheat bran plus barley spent grain at 1 : 1 proportion (18.65 U/gds). In this case, the activity was 3.2-fold higher than that verified with olive oil supplemented wheat bran. The use of olive oil supplemented wheat bran plus citrus pulp improved enzyme production especially at 3 : 2 proportion (14.32 U/gds and 6.35 U/mg of protein), but to a lower level than that with wheat bran plus barley spent grain.

**Figure 1:**
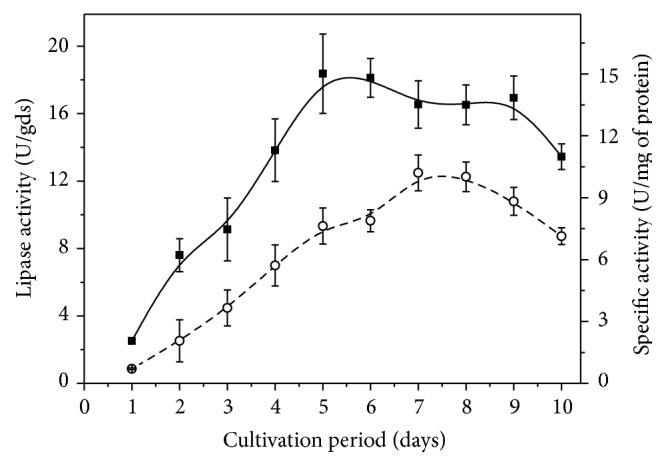
Time course of lipase production by* C. viswanathii* in solid-state cultivation using wheat bran plus barley spent grain 1 : 1 (w/w) supplemented with 25% (w/w) olive oil. Cultures were carried out without nitrogen source supplementation and 50% (w/v) initial moisture provided by Vogel salts solution at 28°C for 120 hours. ■: lipase production (U/gds); ∘: specific activity (U/mg of protein).

Lipase production by* C. viswanathii* using wheat bran plus barley spent grain 1 : 1 (w/w) supplemented with 25% (w/w) olive oil and 50% (v/w) initial moisture was assayed for 10 days (Figure 1). Lipase activity increased up to the 5th day (18.36 U/gds) and slightly decreased up to the 9th day. The highest specific activity (10.20 U/mg of protein) was observed after 7 days of cultivation.

#### 3.1.1. Effect of Triacylglycerols on Lipase Production

**Figure 2:**
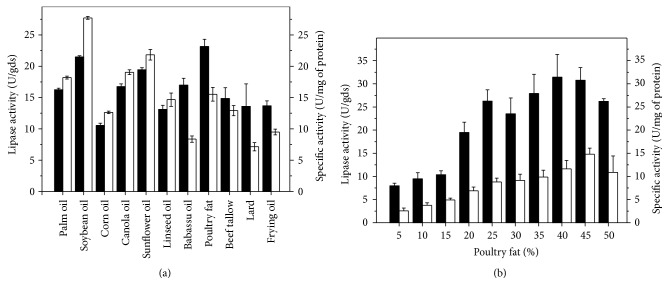
Effects of different triacylglycerol sources (a) and poultry fat concentration (b) on lipase production by* C. viswanathii *in solid-state cultivation. Cultures were carried out with wheat bran plus barley spent grain supplemented with 25% (w/w) of each triacylglycerol source without nitrogen source supplementation and 50% (w/v) initial moisture provided by Vogel salts solution, at 28°C for 5 days (a). Cultures were carried out in the same conditions with only poultry fat (b). ■: lipase production (U/gds); □: specific activity (U/mg of protein).

Triacylglycerols are important inducers of lipase production and in this sense palm oil, crude babassu oil, crude linseed oil, canola oil, sunflower oil, corn oil, soybean oil, and also renewable and low cost sources such as poultry fat, lard, beef tallow, and frying oil were evaluated (Figure 2(a)).* C. viswanathii* strain was able to produce high levels of lipase in medium with all triacylglycerols. Poultry fat induced the highest lipase production (24.20 U/gds), followed by soybean oil (21.49 U/gds) and sunflower oil (19.43 U/gds). The highest specific activity (26.00 U/mg of protein) was detected with soybean oil. Due to these results, lipase production was evaluated at different concentrations of poultry fat (Figure 2(b)). Lipase production increased by increasing poultry fat concentration up to 40% (w/w) corresponding to 31.43 U/gds. Above this concentration enzyme production slowly decreased.

#### 3.1.2. Effect of Nitrogen Sources on Lipase Production

Supplementation with different nitrogen sources improved lipase production by* C. viswanathii* (Table 2). Among them, yeast extract was the best source improving 3.8-fold the lipase production (119.91 U/gds) and also increasing the specific activity (65.50 U/mg of protein). The highest specific activity was verified with whey powder corresponding to 93.53 U/mg of protein.

**Figure 3:**
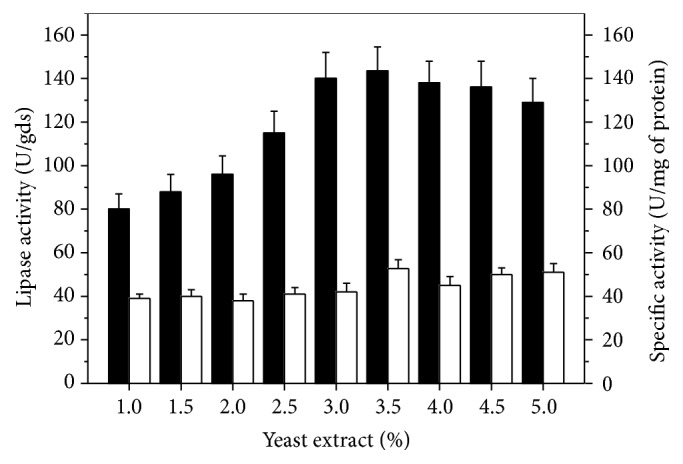
Effect of yeast extract concentration on lipase production by* C. viswanathii* in solid-state cultivation. Cultures were carried out with wheat bran plus barley spent grain supplemented with 25% (w/w) of each triacylglycerol source and 50% (w/v) initial moisture provided by Vogel salts solution, at 28°C for 5 days. ■: lipase activity (U/gds); □: specific activity (U/mg of protein).

The effect of different yeast extract concentrations (Figure 3) showed that lipase production increased by increasing nitrogen source concentration, reaching the highest activity (143.60 U/gds) with 3.5% (w/w) yeast extract and specific activity of 52.85 U/mg of protein.

#### 3.1.3. Effect of Temperature and Moisture on Lipase Production

Cultivation in temperatures from 15 to 40°C showed the highest lipase production of 143.4 U/gds and the highest specific activity (65.8 U/mg of protein) at 30°C (Table 3). Above this temperature there was a reduction in enzyme production to 129.1 and 25.8U/gds at 35 and 40°C, respectively.

The effect of substrate moisture on lipase production revealed optimal production with 40% (v/w) initial moisture (157.3 U/gds), in which the highest specific activity (136.2 U/mg of protein) was also observed. Cultures with 30 or 50% initial moisture resulted in intermediate lipase production of 147.5 and 143.4 U/gds, respectively.

### 3.2. Enzyme Purification

**Figure 4:**
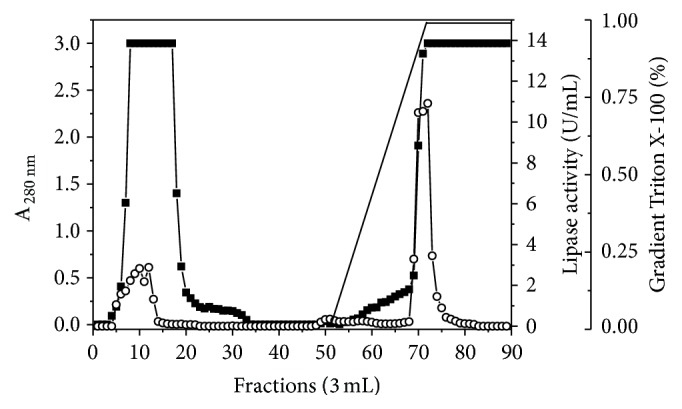
Profile of hydrophobic interaction chromatography of* C. viswanathii* lipase produced in solid-state cultivation under the best conditions for enzyme production. Chromatograph conditions: 0.02 M ammonium acetate buffer pH 6.9; 2.0 mL/min flow rate; 3.0 mL fractions, at 4°C; elution with Triton X-100 0–1.0% (w/v).

**Figure 5:**
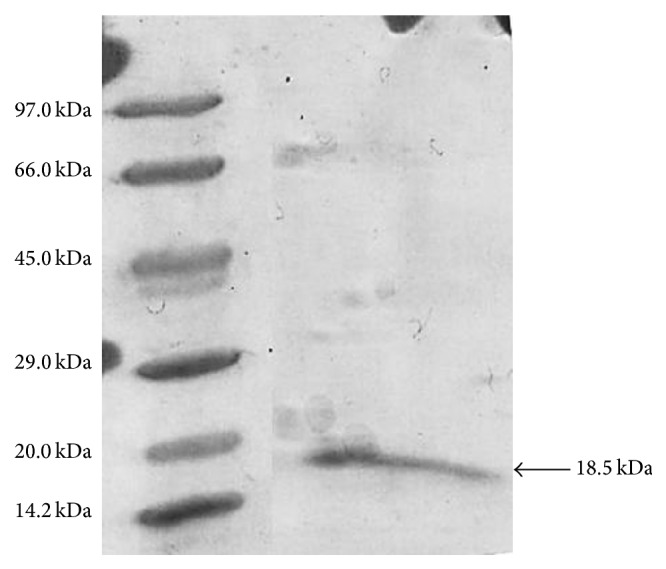
SDS-PAGE of purified lipase from* C. viswanathii* produced in solid-state cultivation. Column 1: standards: phosphorylase b (97 kDa), albumin bovine serum (66 kDa), ovalbumin (45 kDa), carbonic anhydrase (29 kDa), trypsin inhibitor (20 kDa), and *α*-lactalbumin (14.2 kDa). Column 2: purified lipase.

In a previous study, the crude extract obtained in cultivation under optimal conditions was subjected to ammonium sulfate precipitation. In this step, aggregation of proteins was observed obtaining inconsistent results after four attempts of precipitation using different salt concentrations (data not shown). Then, the crude extract was used to select a resin for hydrophobic chromatography. It was observed that the lipase adsorbed in phenyl and Octyl Sepharose without ammonium sulfate; then hydrophobic chromatography was carried out using Octyl Sepharose column without previous salt equilibrium. The crude extract was subjected to dialysis against 0.02 M ammonium acetate buffer pH 6.9 and applied to hydrophobic column equilibrated in the same buffer (Figure 4). Under these conditions, two peaks with lipase activity were observed. The first peak corresponded to nonbound proteins eluted in the initial fractions and the second one to proteins eluted with 1.0% (w/w) Triton X-100 gradient. Fractions with lipase activity in the second peak were pooled and subjected to SDS-PAGE, which showed electrophoretic homogeneity of one 18.5 kDa band (Figure 5). The enzyme was 47.0-fold purified with 133.6 U/mg of protein and the process presented 84.5% yield (Table 4).

### 3.3. Biochemical Characterization

#### 3.3.1. Effect of pH and Temperature on Activity and Stability

**Figure 6:**
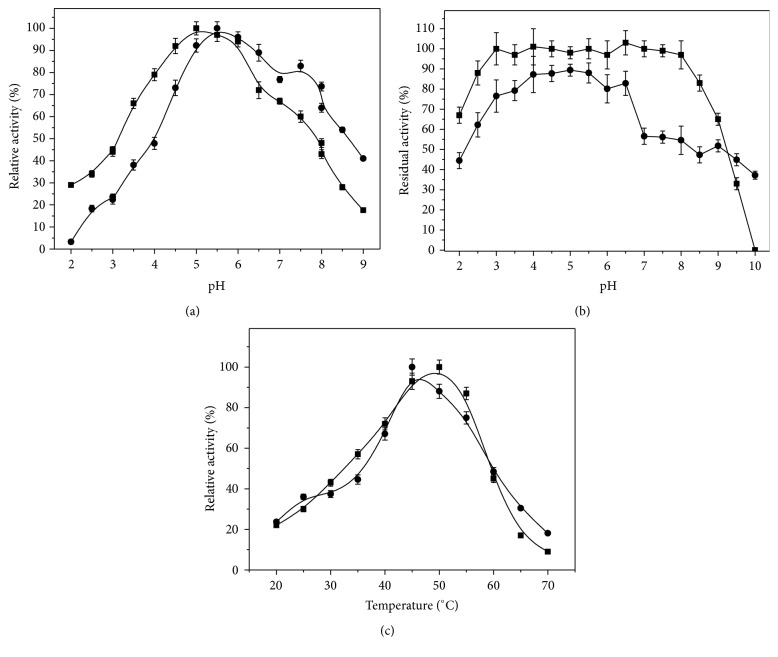
Optimum pH (a) and pH stability (b) and optimum temperature (c) and thermal stability (d) of crude and purified* C. viswanathii* lipase. Assay conditions: 0.05 M glycine-HCl buffer pH from 2.0 to 3.0, McIlvaine buffer pH from 3.0 to 8.0, and 0.05 M glycine-NaOH pH from 8.6 to 10 at 37°C (a); the crude enzyme was incubated in the same buffers for 24 h at 10°C and lipase activity was assayed in McIlvaine buffer pH 5.0 for crude enzyme and 5.5 for purified enzyme at 37°C (b); lipase activity assays were assayed in McIlvaine buffer pH 5.0 for crude enzyme and 5.5 for purified enzyme (c); ■: crude enzyme; ●: purified enzyme.

The effect of pH on the activity of crude and purified lipase was determined from 2.0 to 9.0 (Figure 6(a)). Optimal activity was observed at pH 5.0–5.5 for crude lipase and pH 6.0 for purified lipase. Crude lipase activity was higher in the pH range of 4.5 and 6.0 (92.0 and 94.0%, resp.), and purified lipase presented high activity at pH 5.0 and 6.5 (~90.0–92.0%). Enzyme activity decreased to lower levels from pH 2.0 to 3.5 and 6.5 to 9.0, presenting only 17.6 and 22.9% of activity at pH 9.0 for crude and purified lipase, respectively. Stability of the enzymes was determined after 24 h incubation in buffers of different pH at 40°C (Figure 6(b)). The crude lipase retained approximately 100% of its activity in the pH range from 3.0 to 8.0 and more than 60% at pH 2.0, 2.5, 8.5, and 9.0. However, at pH 9.5 the residual activity was 32.0%, and at pH 10.0, the enzyme completely lost its activity. The purified lipase retained approximately 85% of its activity in the pH range from 4.0 to 5.5. The stability of the enzyme decreased in pH from 6.5 to 10.0 and below 3.5.

The effect of temperature on lipase activity was evaluated from 20 to 70°C (Figure 6(c)). The optimal crude lipase activity was observed at 50°C (100%), and high activities were also observed at 45 (90.5%) and 55°C (86.0%). The activity was about 50% reduced at 35 and 60°C; and low activity was observed at 70°C. The optimal purified lipase activity was 45°C followed by a decrease up to 70°C (19% of activity). Thermal stability of the lipase was evaluated by the incubation of the enzyme without substrate in a buffered medium at pH 5.0 for crude lipase and at pH 6.0 for purified lipase, at different temperatures. The crude* C. viswanathii* lipase retained 97.0% of activity after 24 h at 45°C. The estimated *T*_1/2_ of the enzyme were 23.5, 1.67, and 0.25 h at 50, 55, and 60°C, respectively (Table 5). Purified lipase presented *T*_1/2_ of 6.7, 4.2, and 0.9 h at 40, 45, and 50°C, respectively. At 55 and 60°C the half-lives of purified enzyme were 0.3 h.

#### 3.3.2. Effect of Organic Solvents

The effect of organic solvents on lipase activity is shown in Table 6. The activity of the crude lipase increased in the presence of glycerol (135%), DMSO, propylene glycol (~111%), ethanol (106%), n-hexane, and methanol (~105%). In media containing other organic solvents the activity remained high as in 1-propanol (98%), n-butanol and toluene (~91%), acetone and isooctane (~90%), acetonitrile (86%), and 2-propanol (78%). Purified lipase maintained its resistance to organic solvents since the activity was also increased by DMSO (113%), 1-propanol (111%), glycerol (109%), ethanol (~107%), and methanol (103%) and retained high activity levels in n-hexane, propylene glycol, isooctane, acetonitrile, toluene, acetone, n-butanol, 2-propanol, and xylol.

The stability of crude lipase after 2 h incubation at 30°C was high in media containing DMSO, glycerol, n-hexane, isooctane and ethanol (more than 90%), and propylene glycol (~89%). Intermediate stability was observed with methanol, 1-propanol, acetonitrile, toluene, acetone, 2-propanol, and xylol. Butanol sharply decreased the lipase activity. The purified lipase presented high stability in ethanol (94%). Intermediate values were observed with acetone, 1-propanol, n-hexane, isooctane, glycerol, propylene glycol, DMSO, 2-propanol, acetonitrile, n-butanol, and methanol (69–53%, resp.). Lower stability was observed with xylol (39%) and toluene (25%).

#### 3.3.3. Kinetic Parameters

Substrate hydrolysis reactions were performed for purified lipase with p-NPP (0.0 to 1.0 mM) to determine *K*_*m*_ and *V*_max_. From these values the turnover number (*k*_cat_) and the catalytic efficiency (*k*_cat_/*K*_*m*_) for purified enzyme were calculated. Purified lipase showed *K*_*m*_ 0.12 mM, *V*_max_ 18.3 *μ*mol/mL·min, *k*_cat_ 45.8 s^−1^, and *k*_cat_/*K*_*m*_ of 3.8 × 10^5^ M^−1^ s^−1^.

#### 3.3.4. Substrate Specificity

**Figure 7:**
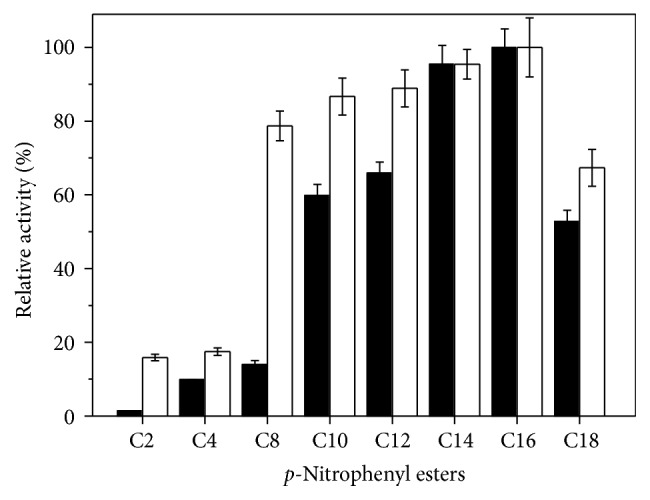
Activity of crude and purified* C. viswanathii* lipase on* p*-nitrophenyl esters. Activity was determined in McIlvaine buffer pH 5.0 at 50°C for crude enzyme and 5.5 at 45°C for purified enzyme. ■: crude lipase; □: purified lipase. C2 acetate, C4 butyrate, C8 caproate, C10 decanoate, C12 laurate, C14 myristate, C16 palmitate, and C18 stearate.

Hydrolytic activity of crude and purified lipase was evaluated on p-nitrophenyl ester substrates (Figure 7). The highest activity of crude and purified lipase was observed with p-nitrophenyl palmitate. The activity of crude lipase increased using esters from decanoate, laurate, and myristate to palmitate; the low activities were observed with acetate, butyrate, and caproate. Activity of purified lipase was high using esters of fatty acids from C8 to C16 and low levels were observed for those of acetate and butyrate. Activity on p-nitrophenyl stearate of crude lipase was lower than on p-nitrophenyl decanoate and for purified enzyme it was lower than on p-nitrophenyl caproate.

### 3.4. Hydrolytic Activity on Triacylglycerols

**Figure 8:**
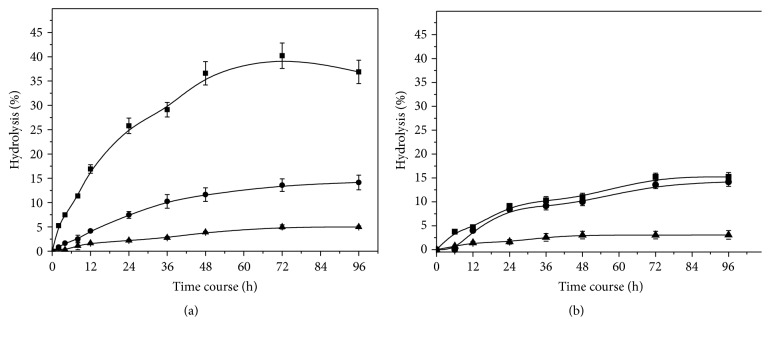
Poultry fat hydrolysis profiles by crude (a) and purified (b)* C. viswanathii* lipase. Assay conditions: hydrolysis was carried out using enzyme concentration 10 U/mL, *S*_0_ = 100 g·L^−1^, 200 rpm at 40°C. ■: pH 4.0; ●: pH 6.0; ▲: pH 8.0.

The hydrolysis of poultry fat and initial hydrolysis rate in different pH values are shown in Figure 8 and Table 7. Higher hydrolytic activity was observed with the crude enzyme at pH 4.0 up to 72 h, reaching 40% hydrolysis (110.9 mmol of fatty acids released) and initial reaction rate of 33.17 mmol/L·min. The slope degree of hydrolysis versus time at *t* = 0  (*dX*/*dt*)_*t*=0_ was 0.0092 and the correlation coefficient (*r*) was 0.905. At pH 6.0 and 8.0 the hydrolytic activity sharply decreased reaching maximal hydrolysis activity of 14.1 and 5.0% at 72 h, respectively. Initial reaction rates under these conditions were 18.75 and 3.60 mmol/L·min, respectively. Purified enzyme presented low hydrolytic activity compared to the crude enzyme. Maximal hydrolysis was also observed at pH 4.0 with 15.2% and initial rate of 7.20 mmol/L·min and *r* ^2^ = 0.845. At pH 6.0 the hydrolytic activity was 14.3% with initial reaction rate of 6.50 mmol/L·min; at pH 8.0 the lowest rate (1.91 mmol/L·min) was observed, reaching only 3.0% of this activity.

## 4. Discussion

Solid-state cultivation for lipase production using yeast cells is a nonconventional practice since the access to nutrients is a limiting factor for this group of microorganisms due to the absence of well-developed hyphae and enzymes production capable of releasing soluble monomers or dimers for microbial nutrition and growth. Substrates such as wheat bran, barley spent grain, cassava peel, sugarcane bagasse, and citrus pulp used for* C. viswanathii* cultivation without or with olive oil supplementation showed wheat bran as the most promising substrate in promoting lipase production. Wheat bran contains soluble sugars (arabinose, glucose, and xylose), protein, and starch providing amino acids, nitrogen, and carbon at adequate proportion to support the production of microbial enzymes, and it is commonly considered an excellent substrate for solid-state cultivation due to its heat dissipation, improved air circulation, and loose particle binding [26, 27].

Mixed substrates formulation of wheat bran plus barley spent grain or wheat bran plus citrus pulp supplemented with olive oil increased the enzyme production by 322 and 174%, respectively, in relation to individual wheat bran, which may be related to an increase in spatial distribution or/and larger surface contact between yeast and substrate during microbial colonization. Benjamin and Pandey [28] observed the highest lipase production by* C. rugosa* using a mixture of fine wheat bran, coarse wheat bran, and thick coconut pie. Growth and yield performances can be improved in relation to traditional submerged systems. Moreover, mixture of different substrates shows better performance by providing a more suitable environment for microbial growth. Edwinoliver et al. [13] developed a mixed substrate containing wheat raw, wheat bran, and coconut oil cake for lipase production by* Aspergillus niger*. A 35% increased yield was observed in comparison to the results from individual substrates. The authors attributed these results to the synergistic effect among the three substrates.

Supplementation of substrates with renewable triacylglycerol such as 25% poultry fat instead of olive oil increased the lipase production to 132%, and at 40% it was increased to 171.2%. This is an important characteristic since olive oil is the most usual inducer in many submerged processes due the high level of oleic acid in its fatty acids composition [29, 30]. Poultry fat is a byproduct of the slaughterhouse with low added value with no application to human nutrition. In addition, it has a high amount of unsaturated fatty acids, such as oleic acid, approximately at 50% [31]. In this point, lipase production in solid-state cultivation by* C. viswanathii* becomes much more attractive since the poultry fat becomes semisolid at room temperature, which can cause problems during submerged cultivation, such as formation of solid aggregates, preventing its use by the microorganism and consequently decreasing lipase production.

Although wheat bran and barley spent grain can provide high quantity of protein, the supplementation with organic nitrogen sources may be better accessed resulting in higher enzyme production. Yeast extract supplementation increased lipase production to 381.5% and when it was employed at 3.5% (w/w) enzyme production increased to 456.9%. Nitrogen sources play an important role in the synthesis of lipase; organic nitrogen sources supply cells with growth factors and amino acids, which are required for cell metabolism and enzyme synthesis [32]. Sun and Xu [33] showed that many organic nitrogen sources, except urea, enhanced lipase production by* Rhizopus chinensis*. Imandi et al. [34] verified lipase production by* Yarrowia lipolytica* using mustard oil cake as substrate was enhanced by supplementation with urea, yeast extract, peptone, and malt extract.

Temperature and moisture are environmental factors that affect microbial growth and enzyme production. Optimum temperature for lipase production by* C. viswanathii* was similar to those observed for other filamentous fungi and yeasts [35–37]. Temperature control is critical for process scaling-up, which cannot be observed in flasks cultivation containing a few grams of substrates, but this is an important factor on a larger scale. The mixture of substrates results in a synergistic effect on enzymes production and prevents the formation of compact mass that can aid in the dissipation of heat generated during microbial growth [3, 38]. Čertik et al. [39] related that many wastes used in solid-state cultivation caused agglomeration of substrate particles creating a more compact mass, which, in turn, interfere with microbial respiration and negatively affect substrate utilization. On the other hand, the addition of nonsieved barley spent grain to the substrates increased product accumulation and also removed heat generated during microbial growth.

*C. viswanathii* produced high level of lipase using 40% initial moisture of the substrates, decreasing after that. Higher moisture content may cause a decrease in substrate porosity, thereby decreasing gas exchange. Lower moisture content can promote low microbial growth and lower degree of expansion of the substrate also decreasing enzyme production [3, 9, 35]. Treichel et al. [30] found that lipase production by* C. rugosa* in solid-state fermentation was optimal using substrates with 70% initial moisture. Similarly, other yeast strains and filamentous fungi also produced high lipase levels with 70% initial moisture [33, 37, 40].

The lipase produced by solid-state fermentation under previous established conditions was purified using hydrophobic interaction chromatography. The procedure was simple involving only one chromatographic step, which did not require ammonium sulfate. Similarly, Bastida et al. [41] related that adsorption in 10 mM phosphate is more than 6-fold faster than immobilization in the presence of 1 M ammonium sulfate. This behavior is different from standard hydrophobic purification protocols in which adsorption rates and process yield strongly increase at high ammonium sulfate concentration. Lipase presents interfacial activation phenomenon in the presence of drops of natural substrates, allowing the adsorption of these enzymes to the hydrophobic interface via very hydrophobic area formed by the internal face of the lid and/or surroundings of the active center [42]. In this sense, by the use of hydrophobic supports somehow resembling the surface of natural substrates and very low ionic strength, lipase becomes selectively adsorbed on these supports via an affinity-like strategy [43].

The purified lipase presented molecular mass of 18.5 kDa, *V*_max_ of 18.3 *μ*mol/min·mL, *K*_*m*_ 0.12 mM, *k*_cat_ 45.8 s^−1^, and *k*_cat_/*K*_*m*_ 3.8 × 10^5^ M^−1^ s^−1^. Microbial lipase usually presents MW between 20 and 90 kDa [44]. Lipase with lower MW was observed in* bacteria* such as* Bacillus licheniformis* (19.2 kDa),* Bacillus subtilis*, and* Bacillus pumilus* (19.3 kDa) [24, 45, 46]. Besides, Brush et al. [47] observed two lipase isoforms from* Ophiostoma piliferum*. The major and the minor lipase from this fungus were copurified by hydrophobic interaction chromatography on Octyl Sepharose followed by ion exchange chromatography on Q Sepharose. The major lipase presented MW of approximately 60 kDa and the minor lipase MW of 5 kDa.

Biochemical characterization of crude and purified lipase is important for further industrial applications in hydrolysis or synthesis reactions. Crude lipase from* C. viswanathii* presented optimum activity at pH 5.0 and maintained above 90% of activity in pH range from 4.5 to 6.0. Optimum temperature was observed at 50°C and the enzyme retained 97% of its activity at 45°C after 24 h of incubation. High stability was observed in the pH range from 3.0 to 8.0. Crude lipase from* C. viswanathii* produced in submerged cultivation presents optimum activity at pH 3.5 and high stability in the pH range from 3.5 to 4.5 and optimum temperature at 40°C, retaining 98% of its activity in this temperature after 24 h incubation [20]. These results show that the cultivation of* C. viswanathii* in solid-state cultivation or submerged cultivation produces enzymes with distinct biochemical properties. Yang and Wang [48] related that the protease and amylase produced by* Streptomyces rimosus* in solid-state cultivation are more stable than those produced in submerged cultivation and the first ones can be temporarily stored without significant loss of activity. These results can be related to the fact that the metabolism exhibited by microorganisms in solid-state cultivation is different from that in submerged cultivation, and the influx of nutrients and efflux of waste materials need to be carried out based on these metabolic parameters [49]. The purified lipase from* C. viswanathii* presented different optimum pH and temperature for activity. Purified lipase was stable in acid pH, but above pH 6.0 the enzyme showed decrease in the activity. The dependence of enzyme activity on pH is a consequence of the amphoteric properties of proteins. Different ionizable groups with different p*K*a values are present on the surface of the protein molecules and surface charge distribution on the enzyme molecules varies with the pH on the environment. These fluctuations in charges may affect the enzyme activity either by changing the structure or by changing the charge of a residue important for substrate binding or catalysis [50]. The removal of proteases and protein fragments frequently present in the crude extract, which in some cases can be considered enzyme-stabilizing substances, can make the enzyme more susceptible to pH changes [51].

The crude lipase was clearly more thermally stable than the purified lipase. Half-lives of the purified lipase were 26.1- and 5.6-fold decreased at 50 and 55°C, respectively. This finding is also observed for the crude and partially purified pectinolytic enzymes from* A. niger* [52]. According to these authors, the thermal stability can change due to the lack of (i) interaction effects among enzyme components; (ii) other proteins besides those components secreted by the organism; and/or (iii) a combination of these two factors.

Lipase stability in organic solvents is an essential prerequisite for its application in organic synthesis, since synthetic reactions with enzymes are often performed in organic media to displace the thermodynamic equilibrium towards synthesis [53, 54]. Solvents were listed according to their hydrophobicity (Log *P*) which ranged from −1.67 to +4.51; negative values indicate that the solvent is water soluble, whereas positive values indicate they are insoluble, causing separation of the aqueous from the organic phase [55]. Lipase has different sensitivity to solvents, but in general, water-miscible polar solvents are more destabilizing than water-immiscible solvents [56]. Nonpolar solvents probably promote changes in the equilibrium between the open and closed conformation of lipase and change substrate solubility and reaction products, while polar solvents are more destabilizing to the protein structure by removing solvation water [57, 58]. Nevertheless, no correlation between Log *P* values of solvents and enzyme stability was observed for crude and purified* C. viswanathii* lipase. Similarly, the lipase from* Rhizopus homothallicus* var.* rhizopodiformis* and* A*.* niger* MYA 135 also shows no correlation between stability and Log *P* values [59, 60]. The high activity and stability of* C. viswanathii* lipase observed in the majority of organic solvents indicate wide application of this enzyme for structured lipid production, biodiesel production, esters synthesis for food industries, and hydrolysis of natural triacylglycerols.

Substrate specificity is important for many industrial applications in food industry and biodiesel production. The crude and purified lipase from* C. viswanathii* showed preference for esters hydrolysis of long-chain fatty acids suggesting that this lipase is true lipase. Fojan et al. [61] related that esterases preferentially break ester bonds of shorter chain fatty acids, while lipase displays much broader substrate range than the esterases. The physical state of the substrate is a probable contributing factor towards the substrate specificity. Long-chain fatty acids are typically insoluble or at least poorly soluble. Thus, the lipase has to be capable of identifying insoluble or strongly aggregated substrates. Since lipase is active towards aggregated substrates, lipase activity is directly correlated with the total substrate area and not with the substrate concentration [61, 62].

Hydrolysis of triacylglycerol is an important industrial operation; the products, fatty acids and glycerol, are basic raw materials with wide range of applications. The fatty acids are used as feedstock for the production of oleochemicals such as fatty alcohols, fatty amines, and fatty esters. In this study, poultry fat was subjected to hydrolysis using crude and purified lipase produced by* C. viswanathii* in three different pH values. Under these conditions, it was observed that the poultry fat hydrolysis at pH 4.0 was 2.84- and 8.0-fold higher than at pH 6.0 and 8.0, respectively. This might be explained by changes in amino acids ionization in the enzyme, which alters the ionic and hydrogen bonds that determine the tridimensional structure of proteins. Purified lipase presented low hydrolytic activity on poultry fat compared to crude lipase. This fact may be related to the absence of ions stabilizing the enzyme structure during the formation of fatty acid-lipase complex. Bengtsson and Olivecrona [63] related that the formation of this complex is considered to be the major factor in product inhibition during triacylglycerol hydrolysis. Bengtsson and Olivecrona [64] reported that the cations of inorganic salts form salts with fatty acids and thus remove them from the oil-water interface. As a result, the availability of the interfacial area to the lipase increases, fatty acid-lipase complex formation remains low, and hydrolysis increases.

The alkaline hydrolysis is the most important current route used for triacylglycerol hydrolysis and it requires acidification of the formed soaps to obtain fatty acids [16]. Energy cost associated with this procedure can be prohibitive turning enzymatic hydrolysis more advantageous. Our results indicate that* C. viswanathii* lipase produced under solid-state cultivation presents optimum activity in acid medium and can be applied for poultry fat hydrolysis under these conditions without acidification of the formed soap to obtain fatty acids.

## 5. Concluding Remarks

This study demonstrated that the lipase from* C. viswanathii* was efficiently produced by nonconventional solid-state cultivation with mixed substrates. High lipase production was obtained from cultivation with wheat bran and spent barley grain at 1 : 1 (w/w) proportion supplemented with 40% poultry fat and 3.5% yeast extract. Low cost poultry fat can substitute expensive olive oil also resulting in high enzyme production. The establishment of physical parameters as temperature and moisture increased fivefold the lipase production. The lipase from* C. viswanathii* was purified to homogeneity electrophoretic using only hydrophobic interaction chromatography, and SDS-PAGE shows MW of 18.5 kDa. Crude and purified* C. viswanathii* lipase showed optimal activity at pH 5.0 and 50°C and at 5.5 and 45°C, respectively; the high stability was observed at pH from 3.0 to 8.0 and at 45°C. The crude and purified enzymes were highly active in the presence of many organic solvents and also presented prolonged stability in a variety of polar and nonpolar solvents, suggesting application of this enzyme in esterification and transesterification reactions. Finally, the crude lipase efficiently hydrolyzed poultry fat in acid condition, indicating that it can be used for this purpose, reducing the costs with the acidification step required to recover the produced fatty acids.

## Figures and Tables

**Table 1:** Lipase production by *C. viswanathii* with agroindustrial wastes in solid-state cultivation.

Substrate	Nonsupplemented		Supplemented with olive oil	
	Lipase activity (U/gds)	Specific activity (U/mg prot)	Lipase activity (U/gds)	Specific activity (U/mg prot)
Wheat bran	0.94 ± 0.10	0.03 ± 0.00	5.79 ± 0.87	3.50 ± 0.20
Barley spent grain	0.40 ± 0.01	0.04 ± 0.00	0.32 ± 0.04	0.37 ± 0.00
Cassava peel	ND	ND	0.17 ± 0.01	1.25 ± 0.10
Sugarcane bagasse	0.10 ± 0.01	0.95 ± 0.00	0.12 ± 0.01	1.04 ± 0.10
Citrus pulp	0.11 ± 0.02	0.01 ± 0.00	0.30 ± 0.04	0.11 ± 0.00
WB + BSG (3 : 1)	0.67 ± 0.03	0.20 ± 0.00	15.82 ± 1.17	6.72 ± 0.70
WB + BSG (3 : 2)	0.88 ± 0.07	0.28 ± 0.00	17.46 ± 1.16	7.94 ± 0.91
WB + BSB (1 : 1)	0.62 ± 0.02	0.33 ± 0.00	18.65 ± 1.77	8.85 ± 0.83
WB + CP (3 : 1)	0.20 ± 0.02	0.04 ± 0.00	9.25 ± 0.90	2.11 ± 0.11
WB + CP (3 : 2)	0.45 ± 0.03	0.17 ± 0.00	14.32 ± 0.78	6.35 ± 0.52
WB + CP (1 : 1)	0.45 ± 0.03	0.18 ± 0.00	10.10 ± 0.55	5.65 ± 0.43

Cultures were carried out for 5 days, at 28°C. Substrates were supplemented with 50% Vogel salts solution without nitrogen source. Substrates with olive oil were supplemented with 25% (w/w) of olive oil. WB: wheat bran, BSG: barley spent grain, and CP: citrus pulp.

**Table 2:** Lipase production by *C. viswanathii* in solid-state cultivation with different nitrogen sources.

Nitrogen sources (5% w/w)	Lipase activity (U/gds)	Specific activity (U/mg prot)
Control	31.43 ± 4.91	11.64 ± 1.81
Yeast extract	119.91 ± 11.68	65.50 ± 6.38
Corn steep liquor	57.96 ± 5.20	49.32 ± 5.35
Whey powder	61.00 ± 4.20	93.53 ± 6.44
Soybean meal	40.65 ± 4.80	24.12 ± 4.03
Soy protein	84.60 ± 4.41	76.22 ± 7.62
Cotton seed protein	57.95 ± 2.83	66.84 ± 3.72

Cultures were carried out on wheat bran plus barley spent grain (1 : 1, w/w), 50% moisture, and 40% (w/w) poultry fat for 5 days, at 28°C. Control was carried out in the absence of nitrogen source.

**Table 3:** Effect of temperature and moisture on lipase production by *C. viswanathii *in solid-state cultivation.

Parameter	Lipase activity (U/gds)	Specific activity (U/mg prot)
Temperature (°C)		
15	26.10 ± 4.42	13.31 ± 1.92
20	56.05 ± 6.07	22.97 ± 2.43
25	77.71 ± 3.36	28.59 ± 2.71
30	143.36 ± 9.65	65.76 ± 3.18
35	129.11 ± 8.15	43.19 ± 2.98
40	25.80 ± 6.17	10.05 ± 1.56

Moisture (%)		
20	98.70 ± 4.87	78.89 ± 3.47
30	147.55 ± 8.43	111.32 ± 4.55
40	157.33 ± 9.25	136.20 ± 5.74
50	143.36 ± 7.47	113.32 ± 6.40
60	137.56 ± 6.34	74.56 ± 5.70
70	92.43 ± 5.78	60.80 ± 4.85

Cultures were carried out using wheat bran plus barley spent grain (1 : 1, w/w), 40% (w/w) poultry fat, and 3.5% (w/w) yeast extract for 5 days (above). Cultures were carried out in the same conditions and at 30°C (below).

**Table 4:** Purification of *C. viswanathii *lipase produced in solid-state cultivation.

Purification step	Enzyme activity (U)	Protein total (mg)	Specific activity (U/mg of protein)	Enrichment	Yield (%)
Crude extract	550.02	193.63	2.84	1.00	100.00
Octyl Sepharose 4CL	465.02	3.48	133.63	47.05	84.55

**Table 5:** Half-lives of crude and purified lipase from *C. viswanathii* produced in solid-state cultivation.

Temperature (°C)	Half-life (h)	
	Crude lipase	Purified lipase
40	n.d.	6.7
45	n.d.	4.2
50	23.5	0.9
55	1.67	0.3
60	0.25	0.3

n.d.: not detected after 24 hours of incubation.

**Table 6:** Effect of organic solvents on crude lipase stability produced by *C. viswanathii* under solid-state cultivation.

Organic solvent	log⁡*P*	Crude enzyme		Purified enzyme	
		Relative activity (%)	Stability (%)	Relative activity (%)	Stability (%)
Control	—	100.0 ± 1.2	100.0 ± 2.0	100.0 ± 2.4	100.0 ± 3.2
Glycerol	−1.67	135.4 ± 4.2	99.5 ± 2.6	109.4 ± 5.7	60.2 ± 2.0
DMSO	−1.38	111.7 ± 4.0	99.8 ± 3.0	113.5 ± 2.5	59.5 ± 3.4
Propylene glycol	−0.92	111.5 ± 3.1	88.7 ± 3.5	97.5 ± 7.7	60.1 ± 3.4
Methanol	−0.76	105.1 ± 3.3	84.6 ± 1.9	103.2 ± 7.5	52.9 ± 6.1
Acetonitrile	−0.40	86.2 ± 1.3	68.1 ± 3.9	88.6 ± 8.2	58.6 ± 2.8
Ethanol	−0.24	106.2 ± 2.2	90.5 ± 4.8	106.8 ± 6.1	94.5 ± 2.1
Acetone	−0.23	90.5 ± 2.2	66.7 ± 2.4	85.8 ± 6.8	69.2 ± 1.8
1-Propanol	0.07	97.9 ± 2.5	72.4 ± 1.3	111.5 ± 9.9	66.9 ± 1.8
2-Propanol	0.25	78.3 ± 2.6	56.0 ± 2.4	83.0 ± 4.3	59.2 ± 1.4
n-Butanol	0.80	92.0 ± 4.0	12.1 ± 2.6	84.1 ± 9.9	58.3 ± 3.5
Toluene	2.50	91.1 ± 3.3	69.8 ± 1.9	87.2 ± 5.3	25.0 ± 2.6
Xylol	3.15	65.9 ± 1.4	51.5 ± 1.3	82.0 ± 7.4	39.1 ± 4.2
n-Hexane	3.50	105.9 ± 2.5	98.0 ± 4.7	98.5 ± 8.6	65.2 ± 3.1
Isooctane	4.51	90.4 ± 2.4	95.1 ± 3.4	91.4 ± 7.8	61.8 ± 2.0

Assay conditions: for stability assays crude and purified lipase were incubated at 30°C at 200 rpm during 2 h and the activity was assayed with p-NPP using McIlvaine buffer pH 5.0, at 50°C. log⁡*P*: logarithm of the partition coefficient (*P*) in octanol/water two-phase system indicates the solvents hydrophobicity. DMSO: dimethyl sulfoxide.

**Table 7:** Parameters of poultry fat hydrolysis using crude *C. viswanathii* lipase produced in solid-state cultivation.

pH	*r*_0_ (mmol·L^−1^·min^−1^)	*r* ^2^
Crude enzyme		
4.0	33.17	0.905
6.0	18.75	0.812
8.0	3.60	0.838

Purified enzyme		
4.0	7.20	0.720
6.0	6.50	0.733
8.0	1.91	0.754
